# Estimation of influenza‐ and respiratory syncytial virus‐attributable medically attended acute respiratory infections in Germany, 2010/11‐2017/18

**DOI:** 10.1111/irv.12666

**Published:** 2019-07-24

**Authors:** Matthias an der Heiden, Udo Buchholz, Silke Buda

**Affiliations:** ^1^ Department for Infectious Disease Epidemiology Robert Koch‐Institute Berlin Germany

**Keywords:** burden of disease, generalized additive model, Germany, influenza, influenza type/subtype, medically attended acute respiratory infection, respiratory syncytial virus

## Abstract

**Background:**

The burden of influenza in primary care is difficult to assess, since most patients with symptoms of a respiratory infection are not tested. The case definition of “medically attended acute respiratory infection” (MAARI) in the German physician sentinel is sensitive; however, it requires modelling techniques to derive estimates of disease attributable to influenza and respiratory syncytial virus (RSV).

**Objectives:**

The objective of this paper was to review and extend our previously published model in order to estimate the burden of RSV and the differential burden of the two influenza B lineages (Victoria, Yamagata) as well as both influenza A subtypes on primary care visits.

**Methods:**

Data on MAARI and virological results of respiratory samples (virological sentinel) were available from 2010/11 until 2017/18. We updated the previously published generalized additive regression model to include RSV.

**Results:**

We found that the proportion of MAARI due to RSV is substantial only in the 0‐1‐ and 2‐4‐year‐old age groups (0‐1 years old: median 7.5%, range 4.0%‐14.8%; 2‐4 years old: median 6.5%, range 4.0%‐10.3%); in the 0‐1 years old age group, RSV leads in almost all seasons to a higher burden than any influenza type or subtype, but this is reversed in the age group 2‐4 years old.

**Conclusions:**

We succeeded in rearranging our previously published model on MAARI to incorporate RSV as well as the two influenza B lineages (Victoria, Yamagata) in the time period 2010 to 2018.

## 1 BACKGROUND

Recently, we published a paper where we estimated the burden of influenza A (H1 and H3) and influenza B in primary care (medically attended acute respiratory infections; MAARI) in Germany from 2001/2002 to 2014/2015.^1^ However, it is increasingly appreciated that influenza B strikes as two different lineages (Victoria, B(Vic), and Yamagata, B(Yam)) which are as distinct from each other as are A(H1) and A(H3). As one consequence, the World Health Organization (WHO) has begun in 2013 to include a virus of both lineages routinely in its recommendations that are updated for each hemisphere on an annual basis.^2^ In accordance, vaccine manufacturers have started to produce quadrivalent vaccines which are already being licensed and used in several countries.^2^, ^3^ On the other hand, the knowledge of the differential burden of the two lineages is more than fragmentary. For example, a recent review of the literature on the burden of influenza B noted that although the “findings suggest that influenza B can pose a significant burden to the global population,” “there are serious gaps in the understanding of the precise magnitude.”^4^ The review did not even attempt to address each influenza B lineage separately. Other recently published estimates did not recognize the specific role of influenza subtypes and lineages.^5^, ^6^

Another major player in the family of respiratory viruses with substantial impact in the winter season is the respiratory syncytial virus (RSV). Both viruses cause a similar syndrome (influenza‐like illness, ILI), but influenza as well as RSV may also present without fever.^7^, ^8^ In Germany, RSV waves may or may not coincide with influenza waves.^9^ To date, few studies have attempted to estimate its burden.^10^, ^11^ With several potential RSV vaccines in the pipeline to licensure^12^, WHO has initiated efforts to establish international RSV surveillance.^7^.

The objective of this paper was to review and extend our previously published model in order to estimate the burden of RSV and the differential burden of the two influenza B lineages (Victoria, Yamagata) as well as both influenza A subtypes on primary care visits.

## 2 METHODS

Data from the German influenza sentinel system were used. Medically attended acute respiratory infections in age groups were weekly reported from around 500 primary care practices. The virological sentinel surveillance is performed by the German National Reference Laboratory for Influenza. From a subgroup of patients, systematic sampling by physicians is done according to the EU ILI case definition. Beside influenza virus detection and differentiation of A subtypes and B lineages, every sentinel sample was analysed also for RSV since 2010.^13^ For excess estimates, we used as a basis the model which we published previously.^1^ Briefly, we described the weekly age group‐specific MAARI attack rate as an additive composition of a periodic baseline, a secular trend and the age group‐specific number of samples tested positive for influenza or RSV multiplied by a season‐specific factor. Since the period baseline as well as the trend might be non‐linear, we used a generalized additive model. To capture the assumed proportionality between the number of positive samples and aberrations in the course of the MAARI attack rate on a timescale of a few weeks, we used a linear link function for the model. In a second step, the number of MAARI attributable to influenza or RSV (irMAARI) was distributed according to the age group‐specific weekly distribution of RSV and influenza subtypes (for influenza A) or lineages (for influenza B).

We made three adaptations to the model: (a) we added RSV data, (b) we separated data on influenza B into the two lineages B(Vic) and B(Yam), and (c) we separated the age group 0‐4 years into 0‐1 and 2‐4 years old. Otherwise, we did not alter the model and estimated first the amount of [influenza + RSV] that exceeds the trend + baseline, and distributed this excess in a second step to the influenza subtypes A(H1), A(H3), B(Vic) and B(Yam) as well as RSV. Finally, we calculated age‐specific attack rates by season and subtype.

## 3 RESULTS

Between 20 and 791 positive samples per age group formed the basis for the modelling work (Table 1). To permit comparison with other studies or countries, Table 1 shows estimated irMAARI attack rates in per cent of the age group (as well as 95% confidence intervals). The season with the highest influenza/RSV impact on the population was season 2017/18, where a total of 11.2% of the population was affected by either influenza or RSV (Table 1), which corresponds to 8.7 million MAARI attributable to influenza (iMAARI) and 575 000 MAARI attributable to RSV (rMAARI) in Germany (Table 2).

**Table 1:** Estimated proportion of population with medically attended acute respiratory infections due to influenza or RSV (irMAARI) by age groups, in % of the age group, (95% CI)

Seasons	Ages (0‐1)	Ages (2‐4)	Ages (5‐14)	Ages (15‐34)	Ages (35‐59)	Ages (60+)	Total
2010/11	10.1 (7.8‐12.5)	13.7 (10.9‐16.5)	10.1 (7.4‐12.6)	3.9 (1.4‐6.4)	2.6 (0.3‐5.1)	0.1 (0‐2)	3.4 (2.3‐4.6)
2011/12	9.2 (7‐11.5)	13.4 (10.8‐15.8)	5.4 (3‐7.8)	0.5 (0‐2.5)	1 (0‐3.1)	0.3 (0‐2)	1.6 (1‐2.6)
2012/13	26.4 (23.6‐29.2)	29.2 (26.5‐31.8)	18.1 (15.1‐21)	8.8 (6.5‐11.3)	10.7 (8.3‐13.3)	3.7 (1.6‐5.9)	9.8 (8.5‐11)
2013/14	4.6 (1.9‐7.4)	10.4 (7.7‐13.1)	4.4 (1.5‐7.2)	0.4 (0‐2.7)	0.8 (0‐2.9)	0 (0‐0)	1.2 (0.6‐2)
2014/15	14 (11.1‐16.9)	22.6 (19.5‐25.5)	13.4 (10.6‐16)	9.2 (7‐11.5)	12.3 (9.9‐14.9)	4.4 (2.5‐6.4)	9.8 (8.6‐11)
2015/16	12.6 (9.7‐15.4)	22.4 (19.7‐25.5)	15.3 (12.6‐18)	6 (3.8‐8.3)	6.8 (4.3‐9.2)	2.1 (0‐4.4)	6.6 (5.4‐7.8)
2016/17	20.1 (17.4‐22.6)	23 (20.1‐25.7)	11.9 (9.6‐14.2)	7.6 (5.4‐9.7)	9.1 (6.9‐11.5)	4.9 (3‐6.9)	8.4 (7.3‐9.5)
2017/18	15.3 (12.5‐18.2)	26 (23.1‐29)	15.8 (13.2‐18.3)	10.1 (7.6‐12.7)	13.5 (11.1‐16)	5.9 (3.8‐8.1)	11.2 (10‐12.5)

**Table 2:** Estimated proportion of population with medically attended acute respiratory infections (MAARI) attributable to influenza types A(H1), A(H3), B(Vic) and B(Yam) as well as RSV

Seasons	RSV	INV	A(H1)	A(H3)	B(Yam)	B(Vic)
2010/11	0.3 (0.3‐0.4)	3.1 (2.0‐4.2)	2.0 (1.3‐2.9)	0.0 (0.0‐0.1)	0.1 (0.1‐0.2)	0.9 (0.6‐1.1)
2011/12	0.3 (0.3‐0.4)	1.3 (0.7‐2.2)	0.0 (0.0‐0.0)	1.0 (0.5‐1.7)	0.0 (0.0‐0.1)	0.3 (0.1‐0.4)
2012/13	0.7 (0.7‐0.8)	9.1 (7.9‐10.2)	3.5 (3.0‐4.0)	2.9 (2.5‐3.3)	2.5 (2.1‐2.8)	0.3 (0.2‐0.3)
2013/14	0.4 (0.3‐0.6)	0.8 (0.3‐1.5)	0.2 (0.1‐0.5)	0.5 (0.2‐0.9)	0.0 (0.0‐0.1)	0.0 (0.0‐0.1)
2014/15	1.2 (1.1‐1.4)	8.6 (7.5‐9.7)	1.2 (1.0‐1.4)	5.4 (4.7‐6.0)	1.9 (1.7‐2.3)	0.0 (0.0‐0.1)
2015/16	0.7 (0.6‐0.9)	5.9 (4.8‐7.0)	2.9 (2.2‐3.5)	0.1 (0.1‐0.2)	0.1 (0.1‐0.2)	2.8 (2.3‐3.2)
2016/17	1.6 (1.4‐1.8)	6.8 (5.9‐7.7)	0.1 (0.0‐0.1)	6.4 (5.5‐7.2)	0.3 (0.2‐0.4)	0.0 (0.0‐0.1)
2017/18	0.7 (0.6‐0.8)	10.5 (9.3‐11.7)	2.5 (2.2‐2.8)	0.4 (0.3‐0.5)	7.5 (6.6‐8.4)	0.1 (0.1‐0.1)

**Figure 1:**
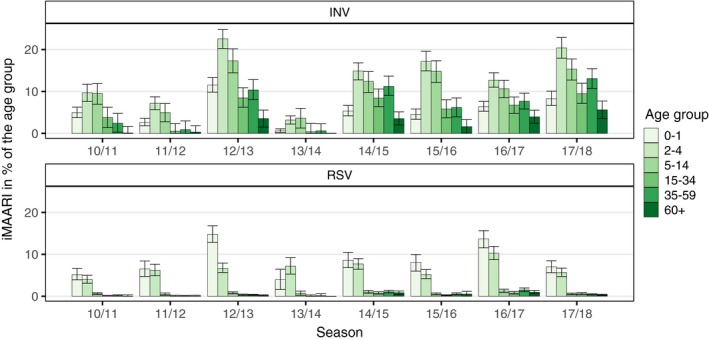
Age‐ and season‐specific attack rate of influenza and RSV‐attributable medically attended acute respiratory infections (irMAARI), in % of the age group with 95% confidence intervals

**Figure 2:**
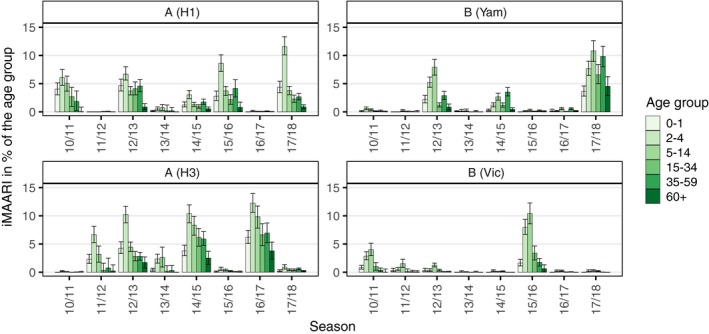
Age‐ and season‐specific attack rate of influenza‐attributable medically attended acute respiratory infections (iMAARI) by subtype/lineage, in % of the age group with 95% confidence intervals

**Figure 3:**
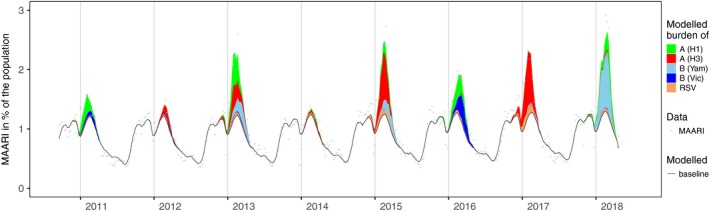
Estimated number of influenza and RSV‐attributable medically attended acute respiratory infections (irMAARI) by influenza type/subtype/lineage and RSV per calendar week (CW 40/2010‐CW 20/2018)

**Figure 4:**
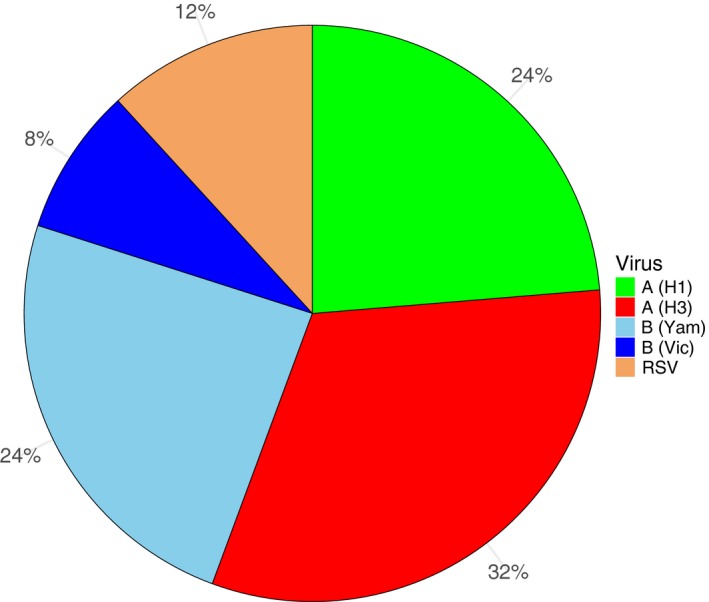
Distribution of irMAARI accumulated for all seasons from 2010/11 until 2017/18, by influenza type/subtype/lineage and RSV

The age distribution of the iMAARI attack rates typically shows a “skewed M” with maxima at the age groups 2‐4 and 35‐59 years old (Figures 1 and 2). In all seasons analysed except season 2013/14, the group aged 2‐4 years old has the highest iMAARI attack rate. In all seasons except season 2010/11, the group aged 35‐59 years old has the highest iMAARI attack rate among the adult age groups (15 years old or older). Regarding B(Yam) and B(Vic), there were three seasons with substantial B(Yam) circulation (2012/13, 2014/15 and 2017/18) and one where B(Vic) circulated strongly (2015/16) (Figures 2 and 3). There was no season with a substantial circulation of B(Yam) and B(Vic) simultaneously (Figures 2 and 3). The respiratory syncytial virus affected all age groups to some degree, but there were only the age groups 0‐1 and 2‐4 years old that experienced a pronounced attack rate (0‐1 years old: median 7.5%, range 4.0%‐14.8%; 2‐4 years old: median 6.5%, range 4.0%‐10.3%), and in 4 of the 8 seasons, it was almost equal in both age groups (Figure 1). In the age group 0‐1 years old, the RSV attack rate was in all seasons higher or equal to the influenza attack rate, and in the age group 2‐4 years old, the RSV attack rate was in all but one seasons lower than the influenza attack rate (Figure 1). For all influenza subtypes or lineages, it could be observed that circulation was generally much less in a season when it followed a season with substantial circulation (Figures 2 and 3). The 8 seasons’ cumulative burden attributable to all four influenza subtypes/lineages and RSV showed that A(H3) had the largest share (32%), followed by A(H1) and B(Yam) with each 24% (Figure 4). The cumulative burden of B(Yam) is thus three times higher of that caused by B(Vic) (8%). The respiratory syncytial virus contributed to 12% of all irMAARI (Figure 4).

## 4 DISCUSSION

In contrast to our previous model where we showed results of estimates of MAARI attributable to influenza A(H1), A(H3) and B, we present now estimates also for RSV and were able to separate B into B(Vic) and B(Yam). The estimated burden in primary care due to the two B lineages reveals that—between 2010/11 and 2017/18—B(Yam) leads to an approximately three times higher burden compared with B(Vic). In a global study with data from 2000 to 2013, Caini found that—among the B lineages—Victoria and Yamagata lineages predominated during 64% and 36% of seasons, respectively. The authors concluded also that the detection of influenza B was more associated with younger age than influenza A. In our analysis, we found that B(Yam) affects also older age groups. During the 2017/18 season in Germany, B(Yam) contributed to the burden of any influenza in an extraordinary manner.

In addition, we were able to estimate the primary care burden due to RSV very well since the underlying data allowed us to analyse separately the 0‐1‐ and 2‐4‐year‐old age groups. Here, we made a number of important observations. First, RSV and influenza waves overlap widely; second, although there is a measurable impact of RSV in all age groups, the proportion of MAARI due to RSV is substantial only in the 0‐1‐ and 2‐4‐year‐old age groups; third, the RSV impact can be observed in these two age groups in all seasons analysed; and fourth, in the 0‐1‐year‐old age group RSV leads in almost all seasons to a higher burden than any influenza, but this is reversed in the age group 2‐4 years old. Because first RSV infection in life affects primarily children under two years of age, these results on RSV were expected and are consistent with study results of the burden of RSV and influenza in secondary care. In comparison with the results of the former model, adding RSV has led to a substantial decrease in the influenza estimate (only) in the 0‐1‐year‐old age group, and the geometrical form of the influenza incidence by age group would now be best described as a “skewed M” instead of a monotonous decline, see Figure 1. The fact that the age group 35‐59 years old was the most affected adult age group might be connected to transmission between children and their parents.

Thus, the inclusion of RSV in the model not only showed the burden of the pathogen in the youngest age group but also improved our estimates for influenza. Opatowski et al stated the important role of considering cocirculating pathogens in mathematical modelling, and to our knowledge, only few studies included both influenza and RSV in burden estimates.^14^, ^15^, ^16^, ^17^

Our model has still some limitations: we continue to have the difficulty that viral samples in the sentinel are taken from ILI patients while we are assessing the total burden of consultations to ARI. Thus, we have assumed that the distribution of INV subtypes/lineages and RSV among ARI cases is similar to that among ILI patients.

We attempted to use the number of influenza and RSV‐positive samples separately in the model. However, we observed that in a couple of seasons the entire burden was taken of by either RSV or influenza in the two youngest age groups. One reason for this was that in seasons with a high MAARI activity already in autumn, this activity can typically not be fully explained by neither RSV nor influenza, and hence, the role of the virus that shows up first in autumn (calendar weeks 40‐52) is overestimated by the model and this is propagated also for the winter (calendar weeks 1‐15) of that season.

We also considered using the positivity rate of influenza or RSV among all tested samples (data and results not shown) instead of the number of positive samples as explanatory variable in the model. However, although the general course was similar, data values of the positivity rate were more erratic, particularly because we considered the age groups separately.

In summary, we extended our burden of disease model for the estimation of MAARI due to RSV in addition to influenza in primary care. This will allow us to monitor the effect of present and future prevention concepts such as vaccination for certain circulating respiratory viruses and to better understand interactions between influenza and RSV.

## References

[1] an der Heiden M , Buchholz U . Estimation of influenza‐attributable medically attended acute respiratory illness by influenza type/subtype and age, Germany, 2001/02‐2014/15. Influenza Other Respir Viruses. 2017;11(2):110‐121. 27754611 10.1111/irv.12434 PMC5304576

[2] Falkenhorst G , Remschmidt C , Weidemann F , et al. Wissenschaftliche Begründung für die Empfehlung des quadrivalenten saisonalen Influenzaimpfstoffs. Epidemiologisches Bulletin. 2018;2:19‐28.

[3] Grohskopf LA , Sokolow LZ , Fry AM , Walter EB , Jernigan DB . Update: ACIP recommendations for the use of quadrivalent live attenuated influenza vaccine (LAIV4) ‐ United States, 2018–19 influenza season. MMWR Morb Mortal Wkly Rep. 2018;67(22):643‐645. 29879095 10.15585/mmwr.mm6722a5 PMC5991811

[4] Paul Glezen W , Schmier JK , Kuehn CM , Ryan KJ , Oxford J . The burden of influenza B: a structured literature review. Am J Public Health. 2013;103(3):e43‐e51. 10.2105/AJPH.2012.301137 PMC3673513 23327249

[5] Hauge SH , Bakken IJ , de Blasio BF , Haberg SE . Burden of medically attended influenza in Norway 2008–2017. Influenza Other Respir Viruses. 2019;13(3):240‐247. 30637942 10.1111/irv.12627 PMC6468058

[6] Tokars JI , Olsen SJ , Reed C . Seasonal incidence of symptomatic influenza in the United States. Clin Infect Dis. 2018;66(10):1511‐1518. 29206909 10.1093/cid/cix1060 PMC5934309

[7] World Health Organization (WHO) . WHO global respiratory syncytial virus surveillance. https://www.who.int/influenza/rsv/rsv_objectives/en/. Accessed February 07, 2018.

[8] Suess T , Buchholz U , Dupke S , et al. Shedding and transmission of novel influenza virus A/H1N1 infection in households–Germany, 2009. Am J Epidemiol. 2010;171(11):1157‐1164. 20439308 10.1093/aje/kwq071

[9] Wadl M , Reiche J , Schweiger B , Köpke K . Do small children with influenza‐like‐illness really have influenza?. European scientific conference on applied infectious disease epidemiology (ESCAIDE) 2008; Berlin, 19‐21112008; Abstract 053, page 144. http://wwwepisouthorg/doc/r_documents/ESCAIDE_Conference_2008_-_Abstract_bookpdf

[10] Fleming DM , Taylor RJ , Lustig RL , et al. Modelling estimates of the burden of Respiratory Syncytial virus infection in adults and the elderly in the United Kingdom. BMC Infect Dis. 2015;15:443. 26497750 10.1186/s12879-015-1218-z PMC4618996

[11] Taylor S , Taylor RJ , Lustig RL , et al. Modelling estimates of the burden of respiratory syncytial virus infection in children in the UK. BMJ Open. 2016;6(6):e009337. 10.1136/bmjopen-2015-009337 PMC4893852 27256085

[12] Mazur NI , Higgins D , Nunes MC , et al. The respiratory syncytial virus vaccine landscape: lessons from the graveyard and promising candidates. Lancet Infect Dis. 2018;18(10):e295‐e311. 29914800 10.1016/S1473-3099(18)30292-5

[13] Buda S , Prahm K , Dürrwald R , et al. Bericht zur Epidemiologie der Influenza in Deutschland Saison 2017/18. Berlin, Germany: Robert Koch‐Institut; 2018 10.25646/5674. https://edoc.rki.de/handle/176904/5739. Accessed on December 19, 2018.

[14] Cromer D , van Hoek AJ , Jit M , Edmunds WJ , Fleming D , Miller E . The burden of influenza in England by age and clinical risk group: a statistical analysis to inform vaccine policy. J Infect. 2014;68(4):363‐371. 24291062 10.1016/j.jinf.2013.11.013

[15] Opatowski L , Baguelin M , Eggo RM . Influenza interaction with cocirculating pathogens and its impact on surveillance, pathogenesis, and epidemic profile: a key role for mathematical modelling. PLoS Pathog. 2018;14(2):e1006770. 29447284 10.1371/journal.ppat.1006770 PMC5814058

[16] Fleming DM , Taylor RJ , Haguinet F , et al. Influenza‐attributable burden in United Kingdom primary care. Epidemiol Infect. 2016;144(3):537‐547. 26168005 10.1017/S0950268815001119 PMC4714299

[17] Jansen AG , Sanders E , Wallinga J , et al. Rate‐difference method proved satisfactory in estimating the influenza burden in primary care visits. J Clin Epidemiol. 2008;61(8):803‐812. 18495428 10.1016/j.jclinepi.2007.08.017

